# Characterization of Nora Virus Structural Proteins via Western Blot Analysis

**DOI:** 10.1155/2016/9067848

**Published:** 2016-05-19

**Authors:** Brad L. Ericson, Darby J. Carlson, Kimberly A. Carlson

**Affiliations:** Biology Department, University of Nebraska at Kearney, 2401 11th Avenue, Kearney, NE 68849, USA

## Abstract

Nora virus is a single stranded RNA picorna-like virus with four open reading frames (ORFs). The coding potentials of the ORFs are not fully characterized, but ORF3 and ORF4 are believed to encode the capsid proteins (VP3, VP4a, VP4b, and VP4c) comprising the virion. To determine the polypeptide composition of Nora virus virions, polypeptides from purified virus were compared to polypeptides detected in Nora virus infected* Drosophila melanogaster*. Nora virus was purified from infected flies and used to challenge mice for the production of antisera.* ORF3*,* ORF4a*,* ORF4b*, and* ORF4c* were individually cloned and expressed in* E. coli*; resultant recombinant proteins purified and were used to make monospecific antisera. Antisera were evaluated via Western blot against whole virus particles and Nora virus infected fly lysates. Viral purification yielded two particle types with densities of ~1.31 g/mL (empty particles) and ~1.33 g/mL (complete virions). Comparison of purified virus polypeptide composition to Nora virus infected* D. melanogaster* lysate showed the number of proteins in infected cell lysates is less than purified virus. Our results suggest the virion is composed of 6 polypeptides, VP3, VP4a, two forms of VP4b, and two forms of VP4c. This polypeptide composition is similar to other small RNA insect viruses.

## 1. Introduction

Nora virus is a picorna-like virus containing a single stranded positive-sense RNA genome ~12,000 bases long. It is related to a few other viruses including an isolate found in the mountain pine beetle, Lotus plant, a parasitoid wasp, and a freshwater mussel [1]. A hallmark of Nora virus infection is the establishment of persistence with little effect on the host [2]. Currently, the virus must be propagated in infected* Drosophila melanogaster* flies as a cell line suitable for its replication is not yet identified.* D. melanogaster* can be infected by a number of viruses. Some are species specific, such as* Drosophila* A, C, and X viruses, Nora virus, and Sigma virus. Other viruses, such as Cricket Paralysis Virus (CrPV), Flock House virus, and Invertebrate Iridescent virus, infect other species of insects, as well as* D. melanogaster* [3]. This makes* Drosophila* a suitable model for virus replication studies.

The Nora virus genome consists of four open reading frames (ORFs). The first three of these overlap each other in alternative reading frames. The fourth open reading frame, based on its position in the genome, appears to be independently read from the other three. Characterization of the virion suggests it is composed of 4 primary proteins, named VP3, and VP4a, VP4b, and VP4c, specified by ORF3 and ORF4 of the viral genome, respectively. The proteins produced from ORF4 are initially translated into a polyprotein and subsequently released by proteolytic processing. In addition, there are several minor protein components of the virion that appear to be derived from these primary proteins [1]. What is not clear is whether these additional polypeptide components are produced by processing events in the infected cell or are artifacts of purification. If they are not artifacts of the purification procedure, then it would be expected that these additional polypeptides would be found in the infected cells of* Drosophila* flies. The objective of this study was to identify the number of polypeptide species that comprise the Nora virus virion by examining the proteins made in infected* D. melanogaster* flies as well as from purified virus utilizing antisera made against whole virus as well as against the individual structural proteins.

Some of the most important aspects of characterizing a new virus are to determine the size, structure, and polypeptide composition of the virion. This involves a number of steps. First, a purification procedure that yields pure virus particles must be established. Second, SDS-PAGE analysis of the purified virus proteins is performed. If the virus is produced in sufficient quantity, Coomassie blue staining is adequate; if not then radioactive labeling of the viral proteins may be needed for detection. Alternatively, antisera can be produced against whole virus particles and the proteins can be detected by Western blot analysis. Antibody reagents have the additional advantage of being useful in virus detection in the infected cell. If the viral genome is sequenced, the predicted viral proteins can individually be cloned, expressed as recombinant proteins, and the recombinant proteins can be used to produce monospecific antisera. Mass spectrometry can also be performed on viral proteins purified from SDS-PAGE gels and compared back to the known nucleotide sequence of the genome to identify viral protein components [1, 4–7].

Characterization of the Nora virus virion shows that two types of particles are readily made in infected* D. melanogaster*. Western blot analyses of proteins from purified virus and from infected cells indicate that the Nora virus virion is composed of 6 proteins, VP3, VP4a, two forms of VP4b, and two forms of VP4c. Additional components of the virion [1] are most likely artifacts of the purification procedure or are produced upon release of the virus particles as final maturation steps.

## 2. Materials and Methods

### 2.1. Flies and Virus

*D. melanogaster* strain* witi Rel* ^*E23*^ infected with Nora virus were a kind gift from Dan Hultmark and Jens-Ola Ekström (Umeå University, Umeå, Sweden). Infected flies were reared under standard conditions at 25°C in a 12-hour light, 12-hour diurnal cycle. The identical uninfected strain was reared under identical conditions in a separate incubator and was confirmed as uninfected by reverse transcription polymerase chain reaction [8]. Infected flies were identified by RT-PCR analysis using primers specific for the VP4a gene (5′-ATGCAGAATCCAACACAAACC and 5′-TCCTAACACCGTTCTCTGTTG). Viral titers in flies were estimated using total RNA isolated from flies and RT-qPCR with these same previously validated primers [2] using a SYBR® Green Quantitative RT-qPCR Kit (Sigma) according to the manufacturer's instructions in a 7500 Real Time PCR System (Applied Biosystems).

### 2.2. Virus Purification and Electron Microscopy

Approximately 3,000 Nora virus infected flies were placed in 10 mM Tris, pH 7.4, and 100 mM NaCl (NT buffer) and homogenized on ice with a motor-driven pestle. The resulting lysate was sonicated on ice for four 15-second pulses with 45 seconds between pulses. The debris was removed by two successive centrifugation steps at 12,000 ×g at 4°C for 20 minutes. The supernatant fractions were pooled and layered onto the top of a cesium chloride step gradient consisting of a 10 mL bottom layer of 1.5 g/mL CsCl and a 10 mL top layer of 1.2 g/mL CsCl. The gradient was spun in a SW28 rotor at 24,000 rpm for 24 h at 20°C. Following the centrifugation of the step gradient, a single virus band appeared at the interface between the two CsCl layers. The virus was recovered from the step gradient using a syringe to puncture through the side of the tube. The crude virus was centrifuged a second time by placing the CsCl solution containing the virus in an SW55 rotor at 28,000 rpm for 24 h at 20°C. Under these conditions, the CsCl forms its own gradient and the virus was separated into two distinct bands, with the virus banding at ~1.33 g/mL (lower band) and 1.31 g/mL (upper band), respectively. The virus bands were harvested independently and dialyzed against 10 mM Tris, pH 8.0, and 250 mM NaCl to remove the CsCl. The virus was stored either at 4°C or at −80°C, depending on the downstream application.

Samples stored at 4°C were sent to the Electron Microscopy Core Facility at the University of Nebraska Medical Center. Grids used for electron microscopy were 200 mesh copper grids coated with formvar and silicon monoxide (Ted Pella, Inc.). A 10 *μ*L drop of each sample collected from the gradient was placed on the grid for one minute. The excess liquid was wicked off with a piece of Whatman 50 filter paper, and the grid was allowed to air-dry for two minutes. A droplet of Nano-Van solution (Nanoprobes, Inc.) was placed on the grid for one minute. The excess liquid was wicked off as above, and the grid was allowed to dry for two minutes. The grids were examined in a Tecnai G2 Transmission Electron Microscope operated at 80 Kv.

### 2.3. RT-qPCR Quantitation of Viral RNA

**Figure 1:**
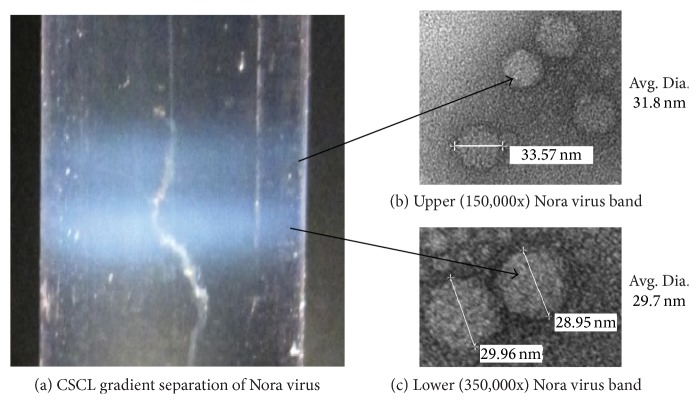
Two distinct sized particles are produced from a Nora virus infection of* Drosophila melanogaster witi Rel* ^*E23*^ strain flies. Approximately 3,000 Nora virus infected flies were homogenized and processed as described in Section 2. In (a) a representative CsCl gradient is shown, depicting the two bands of virus particles obtained. The bands were collected individually and dialyzed against 10 mM Tris-HCl, pH 8.0, and 250 mM NaCl buffer. Electron micrographs of the particles obtained from the upper band (b) and the lower band (c) are shown. The average sizes of the virus particles were 31.8 nm (upper band; *n* = 47) and 29.7 nm (lower band; *n* = 39), respectively.

Viral RNA was quantitated from upper band and lower band virus samples from the isopycnic CsCl gradients (Figure 1) as follows. Virus protein from the two samples was quantitated using the Bradford Assay (BioRad) and equal amounts of virus from the upper and lower bands (5 ng, 2.5 ng, 1.25 ng, 625 pg, and 313 pg) were assayed in duplicate using a SYBR® Green Quantitative RT-qPCR Kit (Sigma) according to the manufacturer's instructions in a 7500 Real Time PCR System (Applied Biosystems). Upper band and lower band virus samples were heated to 95°C for 15 minutes to disrupt particles immediately prior to addition to the RT-qPCR reactions. Data analysis of the RT-qPCR reactions was performed as described by Livak and Schmittgen [9].

### 2.4. Cloning and Expression of Nora Virus Genes

Nora virus gene sequences were obtained commercially (GenScript) and were codon optimized for their expression in* E. coli*. An In-Fusion® HD Cloning Kit (Clontech) was used for cloning optimized genes* ORF1*,* ORF3*,* ORF4a*,* ORF4b*, and* ORF4c*, into the linearized* pET28a* vector and they were transformed into competent Stellar cells (Clontech) per the manufacturer's instructions. Colonies were screened by PCR and colonies testing positive for the various Nora virus genes were grown for plasmid DNA preparation using a Qiagen Plasmid preparation kit. Plasmid DNA was used to transform One Shot® BL21 (DE3) pLysS Chemically Competent* E. coli* cells (Invitrogen). These cells were subsequently used for expression of the Nora virus genes.

Transformed* E. coli* BL21 (DE3) cells were placed in 5 mL of LB-Kanamycin (50 *μ*g/mL) and incubated overnight at 37°C with shaking. Flasks containing 50 mL of LB-Kanamycin broth were inoculated with 500 *μ*L of the overnight cultures and incubated at 37°C until midexponential phase (OD_600 nm_ = 0.5) was reached. Isopropyl *β*-D-thiogalactoside (IPTG) was added to a final concentration of 1.0 mM to each culture. One-milliliter samples were removed from each culture at 0 hours, 1 hour, 4 hours, and overnight following the IPTG addition. Pellets were collected by centrifugation at 13,000 rpm for three minutes at room temperature and resuspended in 200 *μ*L distilled water, and an equal volume of 2x Laemmli sample buffer (BioRad) was added and boiled for approximately two minutes. Twenty microliter samples were run on Mini-PROTEAN® TGX*™* Gels (BioRad) and stained with Coomassie brilliant blue (BioRad). For large scale protein preparations, two milliliters of overnight cultures was placed in 200 mL fresh LB-Kanamycin. Cells were incubated at 37°C until midexponential phase (OD_600 nm_ = 0.5) and IPTG was added to a final concentration of 1.0 mM to each culture and allowed to grow for an additional 24 h at 37°C incubation.

### 2.5. Immobilized Metal Ion Affinity Chromatography (IMAC)

*E. coli* cells were collected from 200 mL cultures (see above) by centrifugation at 8,000 ×g for 10 minutes at 4°C. Supernatant fractions were removed and 10 volumes (g/mL) of lysis/wash buffer (300 mM KCl, 50 mM KH_2_PO_4_, pH 8.0, 20 mM Imidazole, and 6 M Urea) were added to resuspend the pellets. Lysates were sonicated on ice for 15-second pulses with 35 seconds between pulses until a total amount of sonication time of 4 minutes was obtained. Lysates were centrifuged at 12,000 ×g for 10 minutes at 4°C. Supernatant fractions were removed and filtered through a sterile 0.22 *μ*m syringe filter before placing them on the IMAC column.

Purification of his-tagged proteins (rVP3, rVP4a, rVP4b, rVP4c, or rORF1) was performed for each protein individually through separate 1.0 mL IMAC cartridges under denaturing conditions or for all the proteins together through a 5.0 mL IMAC cartridge under denaturing conditions. A column packed with Profinity*™* IMAC (BioRad) resin was equilibrated with 5 column volumes of equilibration/wash buffer 1 (300 mM KCl, 50 mM KH_2_PO_4_, pH 8.0, 20 mM Imidazole, and 6 M Urea). The cartridge was washed with 6 column volumes of both wash buffer 1 and wash buffer 2 (20 mM Tris, pH 8.0, 50 mM KCl, 20 mM Imidazole, and 2 M Urea). Proteins were eluted with 10 column volumes of elution buffer (20 mM Tris, pH 8.0, 50 mM KCl, 10 mM–250 mM Imidazole, and 2 M Urea) and analyzed by SDS-PAGE with Coomassie blue staining. Purified protein samples were pooled for determination of protein concentration using Pierce*™* BCA Protein Assay Kit (Thermo Scientific) following kit instructions.

### 2.6. Preparation of Mouse Antisera against Nora Virus and Recombinant Nora Virus Proteins

All experimental procedures were reviewed and approved by the UNK Institutional Animal Care and Use Committee (IACUC # 091311). Mouse antisera against purified virus or the individual recombinant proteins were prepared as follows. CD-1 outbred retired breeder female mice (Charles Rivers) were used for production of antisera. Mice were housed, 5 per cage, in a holding facility maintained at constant temperature (25°C) under a 12 : 12 light/dark cycle. Food and water were provided* ad libitum*. All mice were prebled via the retroorbital route [10]. Twenty micrograms of virus (lower band) or recombinant protein was mixed with enough 10 mM Tris, pH 8.0, and 250 mM NaCl buffer to give a volume of 100 *μ*L. An equal volume of Freund's complete adjuvant (Pierce Chemical Co.) was added and an emulsion was formed by passing the mixture back and forth through a double-hub needle. Two hundred microliters of the emulsion containing the virus or protein was administered to each mouse via a subcutaneous route on day one. Four weeks later, a test bleed from the mice was taken and a booster injection identical to the primary injection was given, with the exception that the inoculum was prepared using Freund's incomplete adjuvant (Pierce Chemical Co.). Every two weeks following the booster injection, additional test bleeds were taken until the titer waned. In some instances, a tertiary injection was given and additional test bleeds were taken as described.

### 2.7. Western Blot Analysis of Viral Proteins

Viral proteins from whole virus (upper and lower bands), recombinant proteins, or infected cell lysates were run on Mini-PROTEAN® TGX*™* Gels (BioRad) as described above and subsequently transferred to nitrocellulose membranes using a semidry blot format (Trans-Blot Turbo Transfer System; BioRad). Following protein transfer, the membranes were blocked with blocking buffer (5% nonfat powdered milk buffered in Tris-HCl, pH 8.0, 150 mM NaCl, and 0.05% Tween 20 (TBST)). The various antisera, either against whole virus or the individual proteins, were diluted in blocking buffer at dilutions varying from 1 : 500 to 1 : 2000 and incubated with the membranes for at least 1 h at room temperature or overnight at 4°C. Following incubation with the primary antibody, the membranes were washed three times for 10 minutes each with TBST. After washing, the secondary antibody (goat anti-mouse IgG alkaline phosphatase conjugate; Pierce Chemical Co.) was diluted in blocking buffer at 1 : 10,000 and incubated with the membrane for at least 1 h at room temperature. The membranes were washed twice with TBS and developed using NBT/BCIP (Pierce Chemical Co.). Blot development was stopped after several minutes with 2 rinses of distilled water.

## 3. Results

### 3.1. Purified Nora Virus from Infected Flies Produces Two Types of Particles

As a first step to fully characterizing the protein composition of Nora virus, we modified the Nora virus purification procedure [2] to include an isopycnic CsCl gradient that followed the CsCl step gradient (see Section 2). The results obtained from the isopycnic gradient (Figure 1) yielded two virus bands. The lower band (*ρ* = 1.33 g/mL) was previously reported [2]. The upper band (*ρ* = 1.31 g/mL) typically contained less virus particles than the lower band as determined by protein assay. When the particles from each band were observed independently by electron microscopy, the average particle sizes were different. The lower band virus averaged a diameter of 29.7 nm (*n* = 47) and the upper band averaged a diameter of 31.8 nm (*n* = 39).

One potential reason the two particle sizes were observed was the upper band virus particles represented empty capsids. This could result in a larger particle size because the RNA may assist the particles to condense upon assembly as reported for other RNA viruses [11]. It would also account for the difference in density of the upper band particles. Using RT-qPCR on the two particles would provide a means of quantitating the amount of RNA in each band. To this end, virus protein was quantitated via Bradford Assay and equal amounts of protein from the two virus bands were used in RT-qPCR reactions to subsequently quantify the amount of RNA present in the two particle types. Viral RNA (data not shown) is approximately 12-fold more abundant in the lower band particles. This result is consistent with the upper band being primarily composed of empty capsids and the lower band representing complete virions.

### 3.2. Expression and Purification of Recombinant Nora Proteins

**Figure 2:**
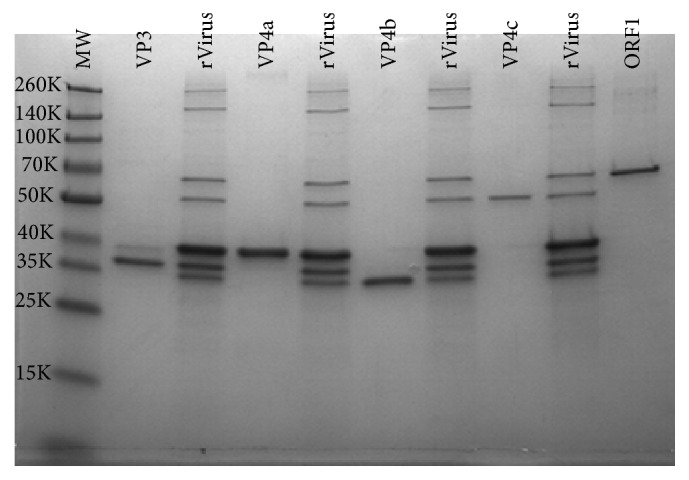
SDS-PAGE analysis of recombinant Nora virus proteins (rNVPs). Recombinant Nora virus proteins were produced by overexpression in* E. coli* cells as described in Section 2. The resultant proteins were purified using Ni^+2^ charged IMAC columns either as individual proteins (lanes labeled VP3, VP4a, VP4b, VP4c, and ORF1) or as a mixture of the 5 proteins (lanes labeled rVirus). The proteins were subsequently separated on 10% SDS-PAGE gels followed by Coomassie blue staining. The results showed the relative purity of the preparations and demonstrated that the proteins readily copurified when mixed together.

**Figure 3:**
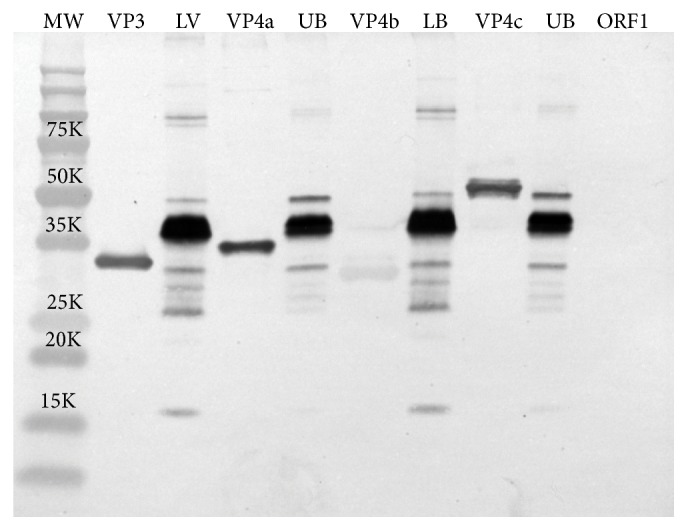
Western blot analysis of Nora virus proteins and recombinant proteins. Mouse antiserum was prepared against whole Nora virus particles (lower band virus particles, see Figure 1) as described in Section 2. Nora virus purified on CsCl gradients was separated into upper band particles (virus UB) and lower band particles (virus LB). The viral protein components of the UB and LB particles were separated on 10% SDS-PAGE gels and blotted onto nitrocellulose membranes, along with the* E. coli* expressed recombinant Nora virus proteins (VP3, VP4a, VP4b, VP4c, and ORF1). The recombinant proteins were either strongly detected (VP3, VP4a, and VP4c), weakly detected (VP4b), or not detected at all (ORF1). There does not appear to be any discernable polypeptide composition differences between the upper and lower band virus particles.

Monospecific antisera to the individual viral proteins would allow the characterization of the protein composition of the two particle types. To accomplish this, each individual viral gene, except* ORF2*, was amplified by PCR from codon optimized genes (see Section 2). After each PCR product was obtained in purified form, they were added to* pET28a* linearized vector and ligated together using an In-Fusion® HD Cloning Kit (Clontech). The subsequent plasmids were purified and transformed into BL21 DE3* E. coli* cells for expression. The proteins were purified from the* E. coli* cultures using the histidine tag added to them by sequences from the* pET28a* vector. The results of the purification are shown in Figure 2. With the exception of VP3, all the viral proteins purified as a single purified species as determined by Coomassie blue-stained SDS-PAGE gels. The lighter band in the VP3 lane appears to be a host* E. coli* protein, as it was not detected by antiserum prepared against whole virus particles (Figure 3). All the virus proteins migrated in accordance with their predicted sizes.

If all five virus proteins were mixed together prior to column purification, they all copurified. This was expected. However, this did result in some higher molecular weight species (~160 K and 260 K) that were not observed from the individual viral protein purifications. We suggest these may be complexes of individual or multiple viral proteins, as they are detected by whole virus antiserum in Western blots (see Figure 3). In addition, these complexes resisted the denaturation steps of SDS-PAGE, indicating a very tight association.

### 3.3. Western Blot Analysis of the Two Particle Types Shows They Are Identical in Protein Composition

The protein composition of the upper and lower band virus particles was further characterized by preparing antisera against a mixture of these particles and subsequently performing a Western blot analysis (Figure 3). In the lanes labeled virus LB and virus UB (lower band and upper band virus particles, resp.) no discernible differences could be observed in polypeptide composition. All the protein species previously reported [1] were detected in both particle types. Therefore, the difference in particle size is again suggested to be from the lack of genomic RNA in the upper band particles.

**Figure 4:**
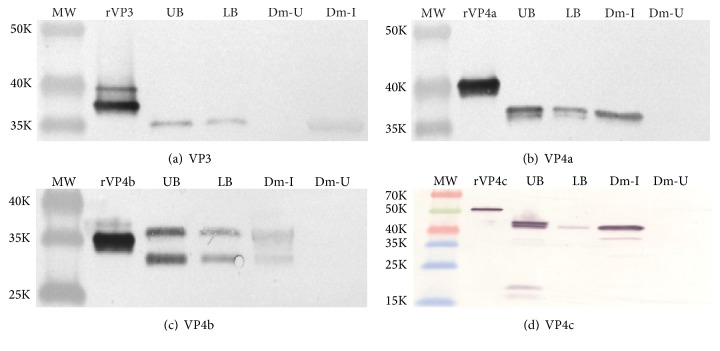
Western blot analysis of the polypeptide composition of the upper band and lower band Nora virus particles using monospecific antisera. Mice were hyperimmunized with rVP3, rVP4a, rVP4b, or rVP4c and the resultant sera were collected as described in Section 2. (a) shows the results of using anti-VP3 antiserum. An SDS-PAGE gel was loaded with molecular weight markers (MW), recombinant VP3 (rVP3), 5.55 *μ*g of upper band virus particles (UB), 5.55 *μ*g of lower band virus particles (LB), Nora virus infected fly lysate (Dm-I), or Nora virus uninfected fly lysate (Dm-U). After electrophoretic separation, the polypeptides were blotted onto a nitrocellulose membrane and developed as described in Figure 4 using a 1 : 1,000 dilution of the primary antibody. (b) (VP4a), (c) (VP4b), and (d) (VP4c) were all run identically to (a) with the exception of the dilution of the primary antibody which was 1 : 500, 1 : 2,000, and 1 : 500, for VP4a, VP4b, and VP4c, respectively.

The antisera made against whole virus particles were shown to react strongly with each of the recombinant-produced proteins, with the exception of VP4b (weak reaction) and ORF1 (no detectable reaction). It is not clear why VP4b gave a weak reaction, as the monospecific sera made against VP4b react very strongly with purified virus (Figure 4). This may indicate that VP4b is not immunogenic or it is not sufficiently exposed in the virion. ORF1 protein was predicted not to react with whole virus antisera as it was not previously reported to be part of the virus particle [1].

### 3.4. Protein Composition of Nora Virus Particles Consists of at Least Six Polypeptides

As a final step to characterize the protein composition of the two types of Nora virus particles, antisera were prepared against each of the recombinant proteins (rVP3, rVP4a, rVP4b, rVP4c, and rORF1). Western blots were developed with each of the antisera (Figure 4). The samples run on each blot were the individual recombinant proteins, upper (UB) and lower (LB) band Nora virus particles, and* D. melanogaster* infected (Dm-I) or uninfected (Dm-U) cell lysates. In each case, the recombinant proteins migrate at a larger size than those from purified virus or infected cells do. This is due to the presence of a HIS-tag on the recombinant proteins. The exception to this is VP4b. The protein from infected cells and purified virus migrated at a larger size than the rVP4b. We suggest VP4b may be posttranslationally modified in some as yet uncharacterized manner, increasing the predicted molecular weight of the protein.

ORF1 monospecific antiserum did not detect proteins in purified virus particles but did detect a single protein species in infected cells (data not shown). This result was consistent with previous reports of Nora virus virion protein composition [1]. As predicted, the VP3 antiserum (Figure 4(a)) detected a single protein species in the virus particles at ~35 K in molecular weight. An identical protein species was detected in infected cell lysate, but not in uninfected cell lysate. The monospecific serum prepared against VP4a (Figure 4(b)) detected two protein species in both virus particle types at ~37 and ~36 K, whereas, in infected cell lysates, only the 37 K protein species was detected. This may suggest that the 37 K protein was processed upon virus assembly or is an artifact of virus purification.  Figure 4(c) shows the results obtained using monospecific antiserum against VP4b. Two protein species were detected, migrating at ~36 K and ~30 K in molecular weight, in both virus particle types and in infected cell lysates. The migration pattern of both species of VP4b suggests both are modified. The 30 K species was previously reported to be derived by a C-terminal proteolytic cleavage from the 36 K species [1]. However, the 36 K species also appears to be modified in some way, as it migrates at a larger size than the recombinant protein (~35 K). The nature of this modification has not been investigated. VP4c antiserum yielded the most complex pattern of the five proteins tested (Figure 4(d)). The upper band virus particles showed at least 5 protein species detected (~42 K, ~41 K, ~35 K, ~20 K, and ~18 K). The blot shown detected only the ~41 K polypeptide in the lower band particles, but in other blots (data not shown) where the NBT/BCIP substrate incubation time was extended to as much as 30 minutes, the other species did stain, but background levels of protein detection in the cell lysate lanes increased dramatically. Two of the protein species were readily detected in the infected cell lysates (~41 K and ~35 K), but the other protein species were not routinely seen. Again, this may be due to processing of viral proteins upon release of mature virus particles, or the additional proteins may be due to an artifact of virus purification. The sizes and numbers of protein species were similar to those previously described [1].

## 4. Discussion

An initial step necessary to understand the function of the protein components of a virus is to determine precisely what proteins are virion components and what proteins specified by the genome are nonstructural proteins. The results of this study have clarified this for the* D. melanogaster* Nora virus. Most insect RNA virus virions are composed of relatively few proteins. For example, CrPV is composed of 4 capsid proteins of molecular weights 33, 31, 30, and 8 kDa [12].* Drosophila* C virions consist of 5 viral proteins, again in the size range of CrPV [13]. Many other RNA viruses of insects also exhibit similar polypeptide composition (reviewed in [14]). The previously reported polypeptide composition of Nora virus [2] suggests a more complex capsid structure consisting of at least 9 polypeptide species ranging in molecular weight from 48 kDa to 17 kDa. The viral genome is predicted to specify 4 structural proteins: one encoded in* ORF3* and the other 3 are derived from* ORF4* via proteolytic cleavage of a polyprotein precursor [1]. This indicated one of two possibilities: either the viral proteins undergo extensive posttranslational processing, primarily proteolytic in nature, or the number of protein species is an artifact of virus purification. If intracellular proteolytic processing was involved, then the protein components found in the mature virions should also be found in the infected cell. On the other hand, if the polypeptide pattern observed in purified virus is an artifact of virus purification, then no such correlation should be found.

The purification procedure for Nora virus was modified from that previously reported [2] to include an extra CsCl gradient step (Figure 1). This yielded upper and lower virus bands of different densities (1.31 and 1.33 g/mL) and sizes (31.8 nm and 29.7 nm). The viral polypeptide composition was identical between the two particles (Figure 3), indicating that the difference in both size and density may lie with the presence or absence of genomic RNA. This was confirmed by RT-qPCR, showing that the lower band contains approximately 12-fold more RNA than does the particle population in the upper band. We suggest that the difference in particle size is due to the viral proteins condensing around the viral RNA in the lower band particles as is known to occur for other RNA viruses during assembly [11]. The detection of two particles that are present as complete virions or empty particles was not unexpected as insect viruses often make much more structural protein than is needed to encapsidate the amount of viral RNA that is replicated (reviewed in [14]).

The production of antisera against whole virus and against the individual viral proteins was used to determine the protein composition of the Nora virus particles. Antibodies against purified virus did not detect ORF1 in virus particles (Figure 3, far right hand lane), nor was it detected in virus particles by monospecific sera against ORF1 protein (data not shown) and therefore this protein is almost certainly not part of the virion. This is consistent with other reports of Nora virus protein composition [1].

Antiserum prepared against VP4a (Figure 4(b)) detected two protein species in virus particles at ~37 and ~36 K, but only the 37 K species was detected in infected cells. This suggests that the 36 K species either is an artifact of virus purification or is derived proteolytically from the 37 K protein during virion release. VP4b was shown previously to exist in two forms [1], a 32 K species and a 26 K species. The 26 K species is a proteolytic cleavage product of the 32 K species. Our results are consistent with this result (Figure 4(c)). Analysis of the amino acid sequence shows a phenylalanine residue 21 amino acids from the C-terminus. This is the cleavage site indicated by Ekström et al. [1] to produce the 26 K species and therefore a chymotrypsin-like protease would be predicted to perform this processing step. The size differential between our results and those reported by Ekström et al. [1] is most likely due to their incorporation of Urea into their SDS-PAGE gels. Unexpectedly, the recombinant protein representing the VP4b sequence migrated at a smaller size than did the virion protein equivalent. This should not be the case as the recombinant protein contains extra amino acids in the form of a histidine purification tag. We therefore suggest that the VP4b species might be modified in some additional fashion during or after translation. Ekström et al. [1] reported the number of proteins originating from the VP4c protein to be at least 5 processed polypeptides. We also detected 5 protein species (Figure 4(d)) of sizes consistent with this observation in purified virus. However, in infected cells, only two protein species were readily detected, the 41 K and 35 K species. Again, this may suggest that the other species derived from VP4c may be an artifact of purification or are produced during virus assembly and release. While analysis of the amino acid sequence of VP4c shows several potential chymotrypsin and trypsin cleavage sites that could yield the 35 K species, without mass spectrometry data, we cannot be certain where the precise cleavage site resides. VP3 monospecific antisera consistently detected only a single viral protein, regardless of whether it was from virus or from infected cells (Figure 4(a)).

## 5. Conclusion

As indicated previously, insect RNA viruses are typically simple in terms of protein composition. We therefore deem it unlikely that Nora virus is composed of such a complex polypeptide mixture. With a particle size of only 31 nm, this would imply a low copy number of each of these proteins, and this would make assembly much more complex than is typical of picornaviruses or picorna-like viruses (reviewed in [7]). We therefore suggest, based on the Western blot results from the infected cell, that the number of proteins composing the Nora virus virion is less than the 9 previously indicated [1] and that some of those protein species are produced by protease action as an artifact of virus purification. The fact that some of the viral proteins, especially VP4c, are susceptible to protease action is demonstrated by the difference in polypeptide composition of virus purified from infected flies versus virus purified from feces [1]. A cell culture system that could be used to propagate Nora virus is desperately needed to be able to investigate these issues further. At the present time, we suggest that Nora virus particles are composed of 6 proteins: VP3, VP4a, two species of VP4b (36 K and 30 K), and two species of VP4c (41 K and 35 K). This polypeptide composition would be more in line with other picornavirus-like RNA insect viruses such as CrPV [12] and* Drosophila* C virus [13]. However, we cannot rule out the possibility that proteolytic cleavage occurs as a result of virus release, making the polypeptide composition of Nora virus virions more complex than these other viruses. Further studies needed to define these maturation events await a cell culture system that is able to propagate Nora virus.
